# Autoimmune Encephalitis in Children: From Suspicion to Diagnosis

**DOI:** 10.7759/cureus.13307

**Published:** 2021-02-12

**Authors:** Kam Lun Ellis Hon, Alexander K Leung, Cheuk Chung Au, Alcy R Torres

**Affiliations:** 1 Pediatrics and Adolescent Medicine, The Hong Kong Children's Hospital, Hong Kong, HKG; 2 Pediatrics, The University of Calgary, Calgary, CAN; 3 Pediatric Intensive Care Unit, Hong Kong Children's Hospital, Hong Kong, HKG; 4 Pediatrics, Boston University School of Medicine, Boston, USA

**Keywords:** autoimmune encephalitis, paraneoplastic encephalitis syndromes, autoantibodies

## Abstract

There are several well-described and studied autoimmune diseases that affect different organ systems, and a limited number of these affect the central nervous system. Autoimmune encephalitis represents a disease with a wide spectrum of clinical manifestations and different levels of severity, from mild cognitive impairment to complex encephalopathy.

Immune-mediated encephalitis refers to a diverse and rare group of conditions in children associated with nonspecific symptomatology, altered mental state, and recalcitrant seizures. Infectious etiology must be excluded. Immune-mediated encephalitis syndromes could be associated with paraneoplastic or primarily autoimmune mechanisms. The newest scientific advantages have concluded that autoimmune encephalitis may be further divided into different groups of diseases depending on the immune response; examples are antibodies to cell surface proteins, antibodies to intracellular synaptic proteins, T-cell response with antibodies to intracellular antigens, among others. Treatment consists of supportive therapy, ranging from supplemental oxygen, fluid restriction to mechanical circulatory support. Specific treatment includes immunoglobulin infusion, plasmapheresis, and pulse steroid treatment. Prognosis is poor if specific treatment is not timely instituted.

The diagnosis of autoimmune encephalitis could be challenging to clinicians due to its diverse clinical features, which can mimic a variety of other pathologic processes. Screening for cancer and proper management that includes immune therapy are fundamental, although some patients will need immune suppression for weeks or months as autoimmune encephalitis may relapse; therefore, follow-up is always necessary.

## Introduction and background

Encephalitis is an inflammatory condition of the brain with multiple etiologies [1-2]. Autoimmune encephalitis syndromes (AESs) describe a basket of encephalitis entities associated with antibodies against the neuronal cell surface or synaptic proteins [2-4]. The target antigens usually play critical roles in synaptic transmission and plasticity. AES may occur in the presence or absence of malignancy, including the classic paraneoplastic encephalitis syndromes associated with antibodies against intracellular neuronal proteins (onconeuronal proteins). Beyond infections, AESs are the largest group of encephalitis in children [5-9].

Clinically, AESs have a wide clinical spectrum that ranges from typical limbic encephalitis to syndromes with complex neuropsychiatric symptoms such as deficits of memory, cognition, psychosis, seizures, abnormal movements, or coma. While patients are often severely affected, these disorders are highly responsive to immunomodulatory therapies. AES is often associated with significant mortality and morbidity [1,6,10-13]. This paper provides an overview of current approaches to the diagnosis and management of autoimmune (antibody-mediated) encephalitis.

## Review

Classification

The exact classification of immune-mediated encephalitides is challenging. Broadly, they are classified into syndromes or according to the exact antibodies (Table 1).

**Table 1:** Types of immune-mediated encephalitis [5,14]

	Paraneoplastic	Auto-antibodies	Miscellaneous
Clinical	Cancer-associated (especially small cell lung cancer (SCLC), limbic system (limbic encephalitis), brainstem (brainstem encephalitis), spinal cord (myelitis), or the entire neuraxis (encephalomyelitis). Acute to subacute onset classic antibody-mediated paraneoplastic encephalitis syndromes include anti-Hu encephalomyelitis (small cell lung cancer, SCLC), Ma2-associated encephalitis (testicular cancer), and anti-collapsin-responsive mediator protein-5 (CRMP5) encephalomyelitis (SCLC or thymoma)	May have tumor-associations, typical limbic encephalitis, syndromes with complex neuropsychiatric symptoms (deficits of memory, cognition, psychosis, seizures, abnormal movements, or coma)	Lupus cerebritis, acute demyelinating encephalomyelitis (ADEM), neurologic systemic manifestations
Imaging	Magnetic resonance imaging (MRI) abnormalities in medial temporal lobes helpful in confirming limbic encephalitis	T2-weighted-fluid-attenuated inversion recovery (T2/FLAIR) hyperintensities of the medial temporal lobes or multifocal T2/FLAIR hyperintensities of the gray matter, white matter, or both, suggestive of demyelination or inflammation	Focal/multifocal lesion(s) predominantly affecting the white matter in acute demyelinating encephalomyelitis (ADEM), cerebral atrophy, small subcortical hyperintensity, and infarct in lupus cerebritis
Laboratory	Antibodies to intracellular antigens (e.g. anti-Hu) T-cells against neurons Inflammation in cerebrospinal fluid	Autoantibodies to extracellular epitopes of ion channels, receptors (e.g. anti- N-methyl-D-aspartate receptor (NMDA) receptor), intracellular synaptic proteins (GAD65)	Lupus anticoagulant, anticardiolipin antibodies, and interleukin 6 (IL-6) in lupus cerebritis
Treatment	Prompt treatment of the tumor and early immunotherapy (glucocorticoids, intravenous immune globulin, plasma exchange, cyclophosphamide, or rituximab) while the neurologic syndrome is still progressing offers the best opportunity to stabilize or slow the progression of the neurologic symptoms.	Corticosteroid, Intravenous immunoglobulin (IVIG), plasmapheresis, rituximab, etc	Corticosteroid, IVIG, plasmapheresis, rituximab, etc
Prognosis	Poor, irreversible neuronal damage	Early treatment (including the tumor, if present) may ensure a better outcome, speedy recovery, and less risk of relapses Reversible effects on synaptic function in neurons	Variable

Antibody-mediated anti-N-methyl-D-aspartate receptor (anti-NMDAR) encephalitis is a classic example of autoimmune encephalitis [14]. Anti-NMDA receptor encephalitis is the most common autoimmune form and is accompanied by ovarian teratoma in 58% of affected women 18-45 years of age [15]. Rasmussen's encephalitis is a rare inflammatory neurological disease, characterized by frequent and severe seizures, loss of motor skills and speech, hemiparesis, encephalitis, and dementia [16]. Affection is usually limited to one hemisphere and generally occurs in children under 15 years. Limbic encephalitis refers to inflammatory disease confined to the limbic system of the brain. The clinical presentation often includes disorientation, disinhibition, memory loss, seizures, and behavioral anomalies. Magnetic resonance imaging (MRI) imaging reveals T2 hyperintensity in the structures of the medial temporal lobes and, in some cases, other limbic structures. Tumor-associated limbic encephalitis is classified according to the autoantibody that causes the disease, including anti-Hu, anti-Ma2, and anti-NMDAR. Once considered an extremely rare disorder, almost always related to cancer and refractory to treatment, limbic encephalitis is now regarded as a relatively frequent disorder, often unrelated to cancer, and with clinical-immunologic variants that respond to treatment [17].

Epidemiology

Epidemiology varies with regions. Autoimmune encephalitis is responsible for a subset of altered mental status previously considered to be idiopathic [18]. The incidence rate of autoimmune encephalitis is estimated to be 0.8/100,000 person-years [19]. The incidence and prevalence of autoimmune encephalitis are higher among African Americans than Caucasians [19]. The prevalence and incidence of autoimmune encephalitis are comparable to infectious encephalitis, and early detection is improving over time. In children, AES is less frequently associated with tumors as compared with adults. Anti-N-methyl-D-aspartate receptor (NMDAR) encephalitis and acute disseminated encephalomyelitis (ADEM) are the most frequently described forms of autoimmune encephalitis in children. In one study of 103 children with AES, 19 children had anti-NMDAR encephalitis and 34 children had ADEM [20].

Etiopathogenesis

ADEM, or acute demyelinating encephalomyelitis, is a rare autoimmune disease that is often triggered by a viral infection and characterized by marked inflammation of the brain and spinal cord [20]. ADEM also attacks the axons of the central nervous system and damages their myelin insulation with resultant white matter injury. Autoimmune encephalitis most common target antigens located in the limbic system [4]. The inflammatory changes produce T2 and fluid-attenuated inversion recovery (FLAIR) hyperintense signal abnormalities in the temporal lobes and limbic structures and T2-weighted hyperintense signal changes on brain MRI [4]. Paraneoplastic disorders are associated with an immune response to antigens in tumor cells and native neuronal cells, which results in an antibody-mediated attack on previously immune-privileged neuronal structures. Paraneoplastic syndromes are most often seen in small-cell lung cancer.

Clinical manifestations

Typically, children with AES are previously healthy and present with acute or subacute onset of neuropsychiatric symptoms [21]. In contrast, the onset is almost always rapid in adults. Prodromal symptoms, such as fever and headaches, occur in over 50% of children [21]. The clinical manifestations of AES are dictated by the specific location of the underlying immune response within the nervous system, which leads to substantial variability. Autoimmune encephalitis is an important cause of new-onset altered mental status, such as memory loss, cognitive dysfunction, and behavioral changes, such as repetitive or stereotypical behaviors; the scope of which has only recently begun to be recognized in the medical literature [2,14]. Clinical manifestations depend on the underlying etiology of the AES and the specific site of involvement and include catatonia, psychosis, abnormal movements, autonomic dysregulation, dysphagia, dysarthria, nystagmus, opsoclonus, vertigo, sensorineural deafness, trigeminal sensory loss, excessive daytime sleepiness, insomnia, tremor, ataxia, dyskinesia, myoclonus, chorea, dystonia, stiff-person syndrome, stupor, confusion, seizures, and coma [4-5,14]. Affected patients may have autonomic dysfunction manifested as bradycardia, tachycardia, dysrhythmia, labile blood pressure, central hypoventilation, and hyperthermia.

Laboratory investigations and imaging

Patients with suspected paraneoplastic or autoimmune encephalitis should have brain imaging, electroencephalography (EEG), lumbar puncture, and serologic testing for appropriate biomarkers to confirm the diagnosis and exclude alternative etiologies.

A brain magnetic resonance image (MRI) is helpful to exclude a cerebrovascular event or metastatic disease. Characteristic MRI findings in patients with paraneoplastic or autoimmune encephalitis include signal hyperintensities on fluid-attenuated inversion recovery (FLAIR) or T2-weighted images in medial temporal lobes and/or brainstem; subcortical regions and the cerebellum are sometimes affected. Contrast enhancement is variable. Although MRI findings are neither sensitive nor specific for these disorders, in the appropriate clinical setting they can be highly suggestive of specific syndromes [22-23].

An EEG should be performed to exclude nonconvulsive seizures in patients with paraneoplastic and autoimmune encephalitis. Nonspecific EEG abnormalities include focal or generalized slowing, epileptiform activity, and lateralized periodic epileptiform discharges (LEDs) previously known as (PLEDs)[24]. Approximately one-third of patients with anti-NMDAR encephalitis have an EEG pattern called extreme delta brush that is considered characteristic of the disorder [24].

Patients with paraneoplastic and autoimmune encephalitis may have normal or abnormal cerebrospinal fluid (CSF) findings. Abnormalities include modest elevation of protein (<100 mg/dL), mild to moderate lymphocytic pleocytosis, elevated immunoglobulin G (IgG) index, and/or the presence of oligoclonal bands [5]. Metabolic and toxic encephalopathies should also be considered and excluded. If the patient does not have a known cancer diagnosis, evaluation for occult malignancy should ensue. Paraneoplastic and autoimmune antibody testing should be performed on both serum and cerebral spinal fluid CSF to avoid false-positive and false-negative results [25]. Broadly, two types of antibodies are involved (Table 2). Identification of these antibodies aids diagnosis, management, and prognostication. However, their absence may not refute the diagnosis. Many more novel autoantibodies are discovered with time and advances in laboratory technologies. Not all of these antibodies or biomarkers have commercially available testing, and some antigens remain to be characterized. Thus, negative results do not exclude a paraneoplastic or autoimmune disorder [25].

Diagnosis

The diagnosis of encephalitis is based on a decreased or altered level of consciousness, lethargy, or personality change for at least 24 hours without any other explainable cause [23]. A definitive etiologic diagnosis may not always be possible [6,13,22,26]. MRI neuroimaging is an essential aspect of evaluation [4,13]. Autoimmune encephalitis remains a diagnosis of exclusion. Recognition of the potential causes of autoimmune encephalitis by the radiologist can be the first step to optimizing clinical outcomes through ensuring that prompt and appropriate clinical workup is performed, including the use of specialized serum/cerebrospinal fluid (CSF) antibody panels, with the ultimate goal of establishing an effective treatment regimen before the onset of devastating complications. The recent consensus statement of the international encephalitis consortium serves as a practical aid to clinicians evaluating patients with suspected encephalitis [13-14,27].

Criteria for diagnosing paraneoplastic disorders and a clinical approach to the diagnosis of the AES have been developed [4,17]. Diagnostic criteria for paraneoplastic limbic encephalitis included (i) short-term memory loss, seizures, or psychiatric symptoms; (ii) <4 years between symptom onset and cancer diagnosis; (iii) exclusion of metastases, infection, metabolic, or other causes; and (iv) one of the following findings: inflammatory CSF changes, electroencephalogram abnormality in the temporal lobes, or temporal lobe T2 or FLAIR hyperintensity on MRI [4,17].

Differential Diagnosis

Beyond viral etiologies, immune-mediated encephalitis is the largest group of encephalitis in children [5-27]. The differential diagnosis of autoimmune or paraneoplastic encephalitis includes encephalitis caused by an infectious agent (e.g., virus, bacteria, fungus, and spirochete) and encephalopathy associated with systemic inflammation (e.g., celiac disease, systemic lupus erythematosus, and sarcoidosis), drug intoxication (e.g., cyclosporine and metronidazole), alcohol intoxication, carbon monoxide poisoning, substance abuse, vitamin B 12 deficiency, metabolic disorders (e.g., mitochondrial disease, leukodystrophies, and Wilson disease), vascular disorders (e.g., reversible posterior leukoencephalopathy syndrome and Susac syndrome), demyelinating and inflammatory disorders (multiple sclerosis, neurosarcoidosis), psychiatric diseases (e.g., childhood disintegrative disorder, conversion disorder, bipolar disorder, and schizophrenia), and in adults, neurodegenerative dementias should be considered (e.g., frontotemporal dementia and Alzheimer disease) [7-9].

Treatment

Tumor screening and treatment are essential to proper management. As autoimmune encephalitis may relapse, follow-up care is important [22]. Patients with autoimmune encephalitis are usually critically ill and managed in the intensive care unit [28]. Immunosuppressive therapy is the cornerstone of treatment. The use of immunosuppressive therapy should be prompt and should not wait for the cancer diagnosis or antibody characterization, provided an underlying infectious etiology has been excluded and there are no other contraindications [28]. The results of antibody testing can then be used to refine or alter the treatment strategy.

The neuronal damage in classical paraneoplastic encephalitis syndromes is T-cell-mediated, rapid-onset, and largely irreversible [29]. Thus, these patients often have limited neurologic recovery even with optimal treatment. The best chance for symptom stabilization or improvement appears to be the early identification and treatment of the tumor and the use of immunotherapy (such as glucocorticoids, intravenous immunoglobulin (IVIG), and plasmapheresis) [28-29]. High-dose corticosteroids are often the first line of treatment and can be given either intravenously or orally [22,30]. IVIG can be used as monotherapy but is more often used after or in combination with high-dose corticosteroid [31]. Likewise, plasma exchange has a synergistic effect when used in combination with corticosteroids [31]. A systematic review of 71 articles (n = 242) on plasma exchange in pediatric anti-NMDAR encephalitis showed a trend toward better outcomes when plasma exchange was administered early and when given in combination with corticosteroids [32]. Cyclophosphamide and rituximab are often used as second-line immunosuppressive therapy. Mycophenolate or azathioprine can be used as a steroid-sparing agent [30].

The autoimmune encephalitis syndromes are often responsive to immunomodulatory therapies, as the associated antibodies are pathogenic and reversibly affect the target antigens [2]. Thus, treatment should aim at antibody depletion and immunosuppression. Specific protocols are not yet validated. Treatment should be individualized taking into consideration the age of the patient, the severity of the disease, and whether an underlying malignancy is present. The response to treatment should be based on clinical evaluation. Seizures should be treated aggressively with antiseizure drugs. Recovery is usually slow but most patients will not require long-term antiseizure drug therapy [2,18]. Powerful immune suppression for weeks or months may be needed in difficult cases. Intensive care support should be provided if necessary.

Prognosis

Early and aggressive treatment for AES leads to better prognostic outcomes [14,18,33]. The various antibodies causing the autoimmune response can result in varying symptoms and respond widely in terms of prognosis (Table 2). About half of patients with anti-NMDAR encephalitis show improvement within four weeks of receiving treatment and 80% of these patients eventually have partial or complete recovery. Some patients took up to 18 months to recover. While anti-NMDAR is the most studied of the antibodies, the treatment for autoimmune encephalitis is generally similar. In some cases, the damage to the brain is irreversible. A 2019 systematic review of 44 research articles (n = 2823) describing prognosis in AES showed that delay in immunotherapy contributed to a variety of worse outcomes for patients with different subsets of AES [34]. No use of immunotherapy, altered consciousness, and intensive care unit admission were variables associated with poor prognosis in anti-NMDAR [34].

**Table 2:** Antibody-mediated encephalitis can also be characterized as either group I or group II according to the location of their neuronal antigens [22,28-29,33,34-36]

	Group I	Group II
Targeting	Intracellular antigens	Cell surface antigens
Underlying malignancy	Use the same cytotoxic T-cell mechanisms when targeting the intracellular neuronal antigens and onconeuronal antigens as part of the immune response to cancer poor clinical outcomes decreased response to immunomodulatory therapy and an increased prevalence of “irreversible” neuronal damage	Less likely to be associated with an underlying malignancy Respond better to early immunomodulatory therapy
Selective examples	Anti-Hu (anti-neuronal nuclear antibody 1) encephalitis: The most common paraneoplastic form relatively poor prognosis associated with small-cell lung cancer features of paraneoplastic encephalomyelitis, paraneoplastic subacute sensory neuropathy, and paraneoplastic cerebellar degeneration. Magnetic resonance (MR) imaging includes T2-weighted-fluid-attenuated inversion recovery (T2-FLAIR) hyperintense lesions in the medial temporal lobes with variable involvement of the cerebellum and brain stem	N-methyl D-aspartate receptor (NMDAR) encephalitis: common subtype in young women and children. IgG against GluN1 subunit of the neuronal NMDAR. well-characterized progression of features characterized by an initial viral-like prodrome (fever, malaise, headaches, and anorexia), followed by psychiatric symptoms (anxiety, depression, schizophrenia, and psychosis), which progress to include temporal lobe dysfunction (amnesia and seizures) and ultimately culminate in severe neurologic deficits, including autonomic dysfunction, dystonia/dyskinesia, and profound encephalopathy relatively good prognosis with early diagnosis and treatment, with complete resolution of neuroimaging abnormalities on follow-up examinations. Normal brain magnetic resonance (MR) imaging findings, or wide variation in the distribution and degree of T2-FLAIR hyperintense signal changes throughout the brain.
	Anti-Ma (Ma1/Ma2/Ma3) encephalitis: better prognosis than anti-Hu strongly associated with testicular tumors in young men and small lung cell carcinoma (SCLC) or breast cancer in older patients combination of limbic, diencephalic, or brain stem dysfunction. the minority had classic symptoms of limbic encephalitis, and the majority with brain stem involvement had ophthalmoplegia Classic T2-FLAIR hyperintense lesions in the medial temporal lobes with variable involvement of the thalamus and brain stem.	Voltage-gated potassium channel (VGKC) encephalitis: common classic features of limbic encephalitis early and prominent development of medically intractable epilepsy. circulating VGKC autoantibodies genetic predisposition to VGKC autoimmunity temporal lobe epilepsy T2-FLAIR hyperintense lesions in 1 or both medial temporal lobes and chronic findings of mesial temporal sclerosis on follow-up imaging antibodies to leucine-rich glioma-inactivated 1, contactin-associated protein-like 2, and dipeptidyl-peptidase-like protein-6
	Anti-CV2 (or collapsin response mediator protein 5) encephalitis: associated with SCLC and malignant thymoma that has prominent T2-FLAIR hyperintense lesions in the striatum clinically resembles choreiform movement disorders less prominent involvement of the medial temporal lobe no restricted diffusion or T2-FLAIR hyperintense lesions in the striatum	VGCC encephalitis: rare subtype described in women and young children classic clinical progression of symptoms from viral prodrome to neuropsychiatric symptoms, limbic dysfunction, seizures extra-limbic involvement with gyriform postcontrast enhancement and cortical laminar necrosis
	Anti-Glutamic Acid Decarboxylase (GAD) encephalitis: Anti-GAD antibodies are not typically associated with malignancy but associated with other non-neoplastic autoimmune conditions such as type 1 diabetes mellitus. Classic temporal lobe lesions on magnetic resonance (MR) imaging with the expected clinical findings of limbic encephalitis plus additional features of stiff person syndrome	γ-Aminobutyric Acid Receptor (GABAr) encephalitis: less common but better overall prognosis subtypes of γ-aminobutyric acid A-receptor or B-receptor subunits γ-aminobutyric acid B-receptor classic features of limbic encephalitis defined by early and frequent seizures with the development of T2-FLAIR hyperintense signal changes in 1 or both temporal lobes seen with small-cell lung cancer or pulmonary neuroendocrine tumors responds well to immunosuppression and removal of the underlying tumor γ-aminobutyric acid A-receptors good prognosis with adequate treatment not associated with cancer extensive T2-FLAIR hyperintense lesions outside of the limbic system

## Conclusions

Autoimmune encephalitis is an inflammatory, rapid, progressive disease that requires extensive and prompt workup. Patients should have neuroimaging, EEG, lumbar puncture, and antibody testing on serum and CSF at the same time that treatment is initiated based on a clinical diagnosis. Further investigation is necessary in search of cancer, central nervous system inflammation, infection, and other alternative etiologies. Early diagnosis and effective treatment might improve long-term outcomes.
